# Simulation Training in a Lower Middle-Income Country: Supporting a New Center and Developing Low-Cost Models for Critical Skill Acquisition

**DOI:** 10.7759/cureus.40950

**Published:** 2023-06-25

**Authors:** Eesha A Irfanullah, Abhishek Chandra, Rafat H Solaiman, Chesney Siems, Sadashivappa Chethan, Kumar Belani, James Harmon

**Affiliations:** 1 Department of Surgery, University of Minnesota Medical School, Minneapolis, USA; 2 Department of Anesthesiology, Narayana Health, Bengaluru, IND; 3 Department of Anesthesiology, University of Minnesota Medical School, Minneapolis, USA

**Keywords:** international collaboration, trauma, low-cost simulation, medical education, simulation, general surgery, international, thoracostomy, pericardiocentesis, low-resource setting

## Abstract

Introduction: There is a demand for surgical simulation training to be made accessible in low-resource countries. We conducted a pilot workshop at a new state-of-the-art simulation center and evaluated two novel low-cost surgical simulation models in a lower middle-income country (LMIC).

Methods: A hands-on workshop to train local educators about simulation training was held at a new simulation center. Participant surveys were analyzed following the “training-the-trainer” workshop. Low-cost, hybrid-fidelity pericardiocentesis and thoracic cavity simulation training models were created using locally available materials. These models recreated the pertinent anatomy at a cost under 20 US dollars each. The models were used to train 109 postgraduate anesthesiology trainees during two hands-on medical education workshops. Participant surveys were collected and analyzed.

Results: Of the local educators who participated in the “training-the-trainer” workshop, 65% “agreed” and 35% “strongly agreed” with the claim that the simulations better prepared the trainees to teach the clinical scenarios. Additionally, 65% of local educators “agreed” and 35% “strongly agreed" that the simulations prepared them to navigate interprofessional care in those scenarios. The low-cost pericardiocentesis simulation was ranked as “good” or “outstanding" by 100% of survey respondents. The low-cost thoracostomy simulation was ranked as “good” or “outstanding” by 64% of survey respondents. Both the pericardiocentesis and thoracostomy simulators were valued for their low-cost design, the recreation of essential anatomy, and immersive design elements.

Conclusion: Our team successfully implemented novel simulators for skill training in an LMIC by working in close collaboration with local experts, with the advancement of local simulation instruction practices. Collaboration is key to increasing access to surgical simulations, particularly in low- to middle-income countries.

## Introduction

There is a continued demand for validated high-quality simulation training and improved training instruments with real-time feedback, particularly for surgical trainees [1,2]. Although it is not a replacement for the bedside clinical skill practice, simulation-based training provides a safe and standardized environment that has been documented to improve clinical performance [2]. Simulation training allows the objective assessment of learners’ performance, individualized coaching, and real-time feedback. Moreover, it is valuable in preparing trainees for rare, emergent, and time-sensitive procedures with potentially serious complications [3]. The 2030 Lancet Commission on Global Surgery highlights the need for safe and effective surgical training in low- and middle-income countries [4]. Globalization, telesimulation, and widespread Internet connectivity have increased educational opportunities for learners in remote locations and broadened the scope and reach of simulation training [5].

Leveraged by long-standing relationships with several prominent medical centers in a lower middle-income country (LMIC), our team assisted in developing a new, high-fidelity, 9,000-square-foot comprehensive simulation center. The simulation center was developed by Bangalore Medical College and Research Institute, Bengaluru, India, and funded by the local government. Center construction was completed within one year, in collaboration with local leadership, with funding from the local government. This new center accommodates a large volume of simulation training and is supervised by full-time simulation staff and clinical educators. The center was designed to address local unmet needs in simulation training. It includes emergency stabilization bays, operating theaters, neurosurgical suites, dedicated labor and delivery rooms, and simulators for ultrasound-guided endoscopic and laparoscopic procedures. At the completion of the center, our visiting simulation team conducted a “training-the-trainer” workshop with hands-on training, along with didactic and interactive debriefing sessions.

Unfortunately, high-fidelity models are not always readily available at all training locations. Therefore, we developed two low-cost, moderate-fidelity, interactive training models that were used in workshops for postgraduate cardiac anesthesia trainees. These models were created for ultrasound-guided pericardiocentesis, and open thoracostomy - two high-stakes and technically challenging emergency procedures. Commercially available simulation training models for both procedures are often expensive and lack interactive elements. Low-cost simulators reported by other authors for ultrasound-guided pericardiocentesis and open thoracostomy have shown promising results, highlighting the value of low-cost simulators for teaching these high-acuity procedures [6-10]. Lord et al.’s 3D-printed low-cost pericardiocentesis model was well-received by emergency medicine residents in the United States, with 92% of the residents “strongly agreeing” that the models effectively mimicked the clinical scenario [6]. Netto et al.’s low-cost porcine model for chest tube insertion was similarly well-received by medical trainees in Brazil, with trainees awarding the simulation an average quality score of 8.8 out of 10 [8]. The simulators we designed were low-cost and derived from readily available plant-based materials. Our low-cost simulators were supplemented with live volunteer “patients” to facilitate immersive clinical scenarios during the workshops. Workshops for the low-cost pericardiocentesis and thoracostomy models were conducted during Continuing Medical Education (CME) Conferences held at Narayana Health City.

Our report illustrates how shared enthusiasm for simulation center development, awareness of training goals, and use of local materials to create novel hybrid models can lead to successful international collaboration in medical simulation training.

## Materials and methods

Training local educators

We supported the development of a new state-of-the-art simulation center in an LMIC. After the development of the simulation center, we collaborated with local medical educators in hosting a training workshop with both didactic and hands-on components for local educators at the new center. The objective of this workshop was to train local educators on how to best utilize the simulation center resources to train future medical trainees. This "training-the-trainer" workshop took place over three days. On the first day, the local educators underwent training regarding designing simulation scenarios for skill training. This was followed by two days of interactive seminars and simulation training. On the first day of simulation training, local educators served as actively enrolled trainees in the various simulation scenarios. On the final day, local educators were required to teach the same simulation sessions to volunteer medical trainees. The simulation scenarios relevant to local healthcare problems included anaphylaxis management, toxin overdose, and trauma triage. Skills emphasized during the training included airway management, advanced cardiac support, and establishing vascular access. The workshop concluded through collective feedback discussions in which participants were surveyed using a 5-point Likert scale. The survey findings were de-identified, aggregated, and analyzed. Course-assigned data collection does not classify as human subjects research. IRB approval was not required by our institution.

Design of simulation models

**Figure 1:**
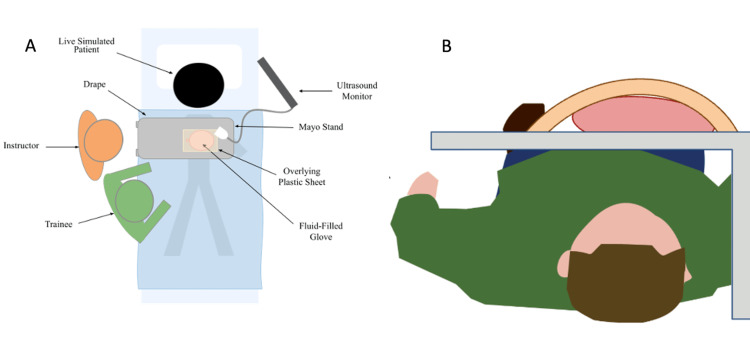
Original illustration of ultrasound pericardiocentesis model (A) Aerial view and (B) transverse view. The protective Mayo stand (gray) was placed over a live supine volunteer. Above the tray, a fluid-filled glove was surrounded by a plastic sheet. The fluid was aspirated from the glove under ultrasonographic guidance. Image credits: Authors' original

**Figure 2:**
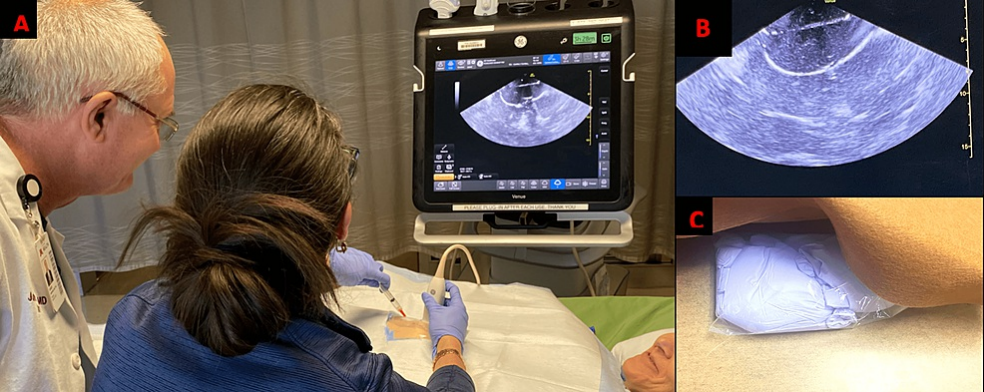
Photographs of the ultrasound pericardiocentesis model (A) A surgical educator guiding a trainee through ultrasound-guided pericardiocentesis with hybrid simulation setup using a volunteer mock patient. (B) Displayed ultrasound image from the simulator design. (C) Simulator construction with skin and drape reflected revealing the “pericardial fluid collection”.

Two low-cost hybrid models were developed for simulation training in an LMIC. A pericardiocentesis training model was designed to accommodate a hands-on workshop using the available hospital equipment and materials. The model construction included a thin elastic latex sheet, surgical gloves, red food coloring, a 30-cc syringe, a 16-gauge angiocatheter needle, intravenous tubing, a collection bag, a malleable aluminum sheet, and a bedside ultrasound machine. The space between the two surgical gloves was filled with an artificial blood mixture to simulate the serosanguinous fluid in the pericardial sac. An elastic latex sheet was placed over the glove to facilitate ultrasound examination and simulate the patient’s skin. This model was placed on a Mayo stand and positioned over the lower thorax and upper abdomen of a supine living volunteer on a gurney as seen in Figure 1 and Figure 2. Replacement components (simulated blood and latex gloves) were required for each participant, and participants were introduced to the clinical scenario through a 10-minute didactic presentation. The pathophysiology of cardiac tamponade, diagnostic clinical features, ultrasound findings, and appropriate procedural steps were reviewed. Critical teaching points of the simulation session are outlined in Table 1. Live volunteers provided an opportunity for an interactive simulation experience, and the trainee maintained a therapeutic alliance with the living volunteer during the ultrasound-guided aspiration of pericardial fluid. Trainees were supervised by clinicians who ensured correct needle placement and Seldinger’s advancement of a pericardial drain.

**Table 1:** Procedural teaching points for ultrasound pericardiocentesis simulation

Procedural step	Critical teaching points
Diagnosis of cardiac tamponade	Interview patient to confirm symptoms and review ultrasound imaging
Initial needle insertion	Use ultrasound to guide approach into the pericardium
Small-volume fluid aspiration from the pericardium	Perform diagnostic aspiration, characterize fluid type, stabilize needle, and prepare to pass the guidewire
Passage of the guidewire	Gently pass the guidewire under ultrasound guidance
Placement of the triple lumen catheter	Gently pass the soft catheter
Attachment of the drainage container	Suture the catheter to the chest wall

**Figure 3:**
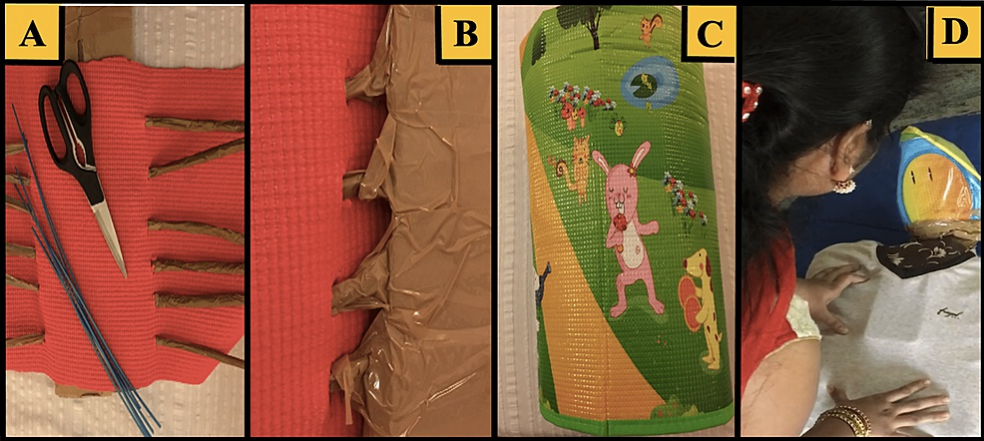
Development of the open thoracostomy training model (A) Yoga mat (red) and plastic brush struts (brown) were constructed to simulate the ribs, spine, and chest wall. (B) Skeletal structures were secured with the packaging tape. (C) The foam pad (green) surrounded a plastic-wrapped pillow to simulate the subcutaneous tissue, pleural space, and lungs. (D) A t-shirt covered the simulated sternum made of cardboard that had an attached cell phone allowing for an educator to provide interactive voice responses as the simulated patient.

Additionally, an open thoracostomy chest tube placement training model was designed using the available hospital equipment and locally sourced materials (Figure 3). The model was constructed using a thin sheet of polyethylene overlying a 1-cm-thick foam to simulate the skin and subcutaneous tissue. The underlying skeletal structures were simulated using polyvinyl chloride plastic. The pulmonary visceral pleura and potential space were simulated using a pillow wrapped in polyethylene to represent the lung and pleural space topographies. This model allowed for the true-to-life replication of rib palpation and haptics during chest tube insertion. A t-shirt was placed over the entire model, and during the simulation, a smartphone was placed within the simulated thoracic cavity for the surgeon educator to remotely communicate with the student during the clinical assessment and to provide interactive voice responses as the simulated patient. The simulated patient was able to provide a limited history and communicated symptoms of chest pain and shortness of breath to the trainee. The participants were introduced to the clinical scenario through a brief didactic presentation. The trainees were expected to diagnose and treat tension pneumothorax using the steps outlined in Table 2. The trainees completed an evaluation of their training experiences for both new models, and their responses were de-identified, aggregated, and analyzed.

**Table 2:** Procedural teaching points for thoracostomy simulation

Procedure step	Critical teaching points
Diagnosis of tension pneumothorax	No breath sounds, hyperresonance, absent sliding sign on ultrasound
Needle decompression	Appropriate orientation: midclavicular line, third rib interspace; angiocath needle is used to decompress
Palpation and identification of landmarks	5th interspace mid-axillary line
Transverse incision and blunt dissection	Administer 1% lidocaine at skin level, intercostal muscles, periosteum rib, and parietal pleura. Make a 2-cm transverse incision and bluntly dissect to the parietal pleura
Dissection and entry into the chest cavity	Dissect up and over the 6th rib into the 5th interspace to avoid injury to the neurovascular bundle
Finger sweep	Explore the chest cavity with finger to assure separation of the parietal and visceral pleura
Chest tube placement in the pleural space	Direct the chest tube appropriately and rotate to avoid kinking
Securing the chest tube	Use heavy sutures to approximate the incision and securely attach the chest tube
Apply dressing	Follow sterile technique
Pleural vacuum	Set to 20 cm of water suction

## Results

**Figure 4:**
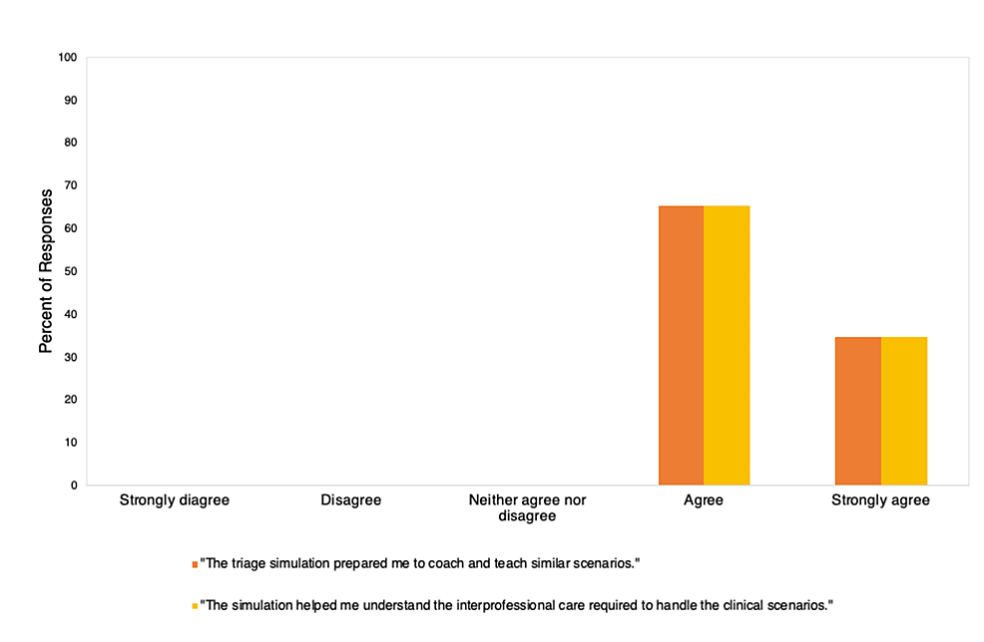
Results from participant evaluations of our “training-the-trainer” workshop

Survey respondents following the “training-the-trainer” workshop (n=95) agreed (65%) or strongly agreed (35%) that the triage simulation prepared them to manage similar clinical scenarios. Survey respondents also agreed (65%) or strongly agreed (35%) that the simulation practice helped them understand the interprofessional care required to handle the clinical scenario. These results are displayed in Figure 4.

**Figure 5:**
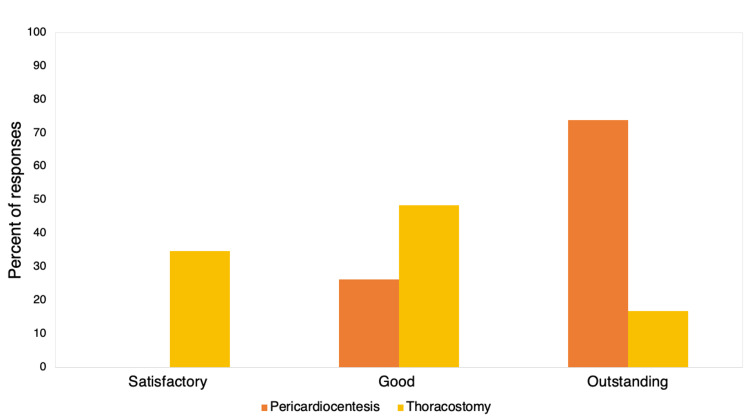
Results from participant evaluations of low-cost model simulation scenarios

A total of 109 participated in the pericardiocentesis and chest tube placement model workshop. For the pericardiocentesis model, 77% (n=84) of participants completed a Likert survey. Trainees rated their experience as “outstanding” (74%, n=62) or “good” (26%, n=22). For the chest tube model, 87% (n=95) of participants completed the survey. The simulation experience was ranked as “outstanding” by 16% (n=15), “good” by 48% (n=46), and “satisfactory” by 35% (n=33). The low-cost model workshop results are summarized in Figure 5. The cost of the materials for the pericardiocentesis and chest tube models were 5 and 20 USD, respectively. The simulators lasted for the entire session and remained intact for future use. The external glove in the pericardiocentesis model needed to be replaced a few times during the workshop to prevent leakage of the red dye (simulated pericardial fluid). Participants and local faculty praised the simulators for their low-cost design, interactive elements, and ability to simulate the pertinent anatomy.

## Discussion

Simulation training is frequently used in medical education to develop competency by teaching critical steps of high-risk procedures in controlled, low-risk environments. Advancements in surgical simulation and training instruments address instructional needs in high-resource countries [11,12]. There is a rising demand for simulation training to be made accessible in low- and middle-income countries. Our team successfully collaborated with our partners in an LMIC to develop multiple practical and sustainable tools for simulation training.

Simulation centers have become increasingly common at academic institutions in high-resource countries; however, these centers are limited in LMICs [1]. Qayumi et al. compiled a list of the primary strengths of and barriers to surgical simulation based on an international survey of 42 simulation centers [1]. The study by Qayumi et al. found there to be three essential characteristics to ensure the success of a simulation center: generous public funding, dedicated simulation technicians, and strong support from institutional leaders [1]. The survey results also demonstrated that a lack of cultural support, as well as, disparities in resources and infrastructure, challenged the success of simulation centers in LMICs [1]. The newly developed simulation center described in our report successfully garnered public funding, dedicated simulation faculty, and institutional support. Our team ensured local support and cultural readiness by working in close collaboration with local experts to help develop the new center.

In a review of American College of Surgeons' accredited simulation centers, Cooke et al. identified four consistent best practices: (1) continuous refinement of curriculum development processes, (2) a standardized process for onboarding new faculty, (3) systematic processes for simulator selection, and (4) adequate and sustained institutional support, all of which were goals adapted by the new simulation center in our report [13]. Our “training-the-trainer” workshop addresses the second of these best practices. While the success of our “training-the-trainer” workshop indicates the promise of this newly developed simulation center, further evaluations of center performance will be necessary to ensure continued adherence to best practices. Such evaluation could not be included in this study due to limitations imposed by the COVID-19 pandemic.

Although high-tech simulation centers are crucial for establishing longitudinal and sustainable improvements in medical and surgical training internationally, the development of these centers is expensive and time-consuming. Additionally, high-fidelity models have not been proven to provide a significant advantage over low-fidelity models in achieving educational outcomes [14]. Low-cost models are valuable options to improve competence without jeopardizing patient safety or draining limited hospital resources [4]. Our innovative pericardiocentesis and thoracostomy simulators offer a heightened level of fidelity through real-time simulated patient interaction while maintaining accessibility and durability.

Ultrasound pericardiocentesis and thoracostomy are both high-acuity, low-occurrence procedures for which trainees can benefit significantly from simulation training. Several simulation models for thoracostomy tube placement and pericardiocentesis have been described in the literature, highlighting the importance of simulation training for these procedures [6-10,15]. A major limitation of simulation training is the cost of each model. Standard market prices for pericardiocentesis and thoracostomy models range from 1000 to 5000 USD. Our pericardiocentesis and thoracostomy models cost only 5 and 20 USD, respectively.

The pericardiocentesis model demonstrated several benefits for low-resource settings in comparison to other models documented in the prior literature. Lord et al. and Tsai and Seslar used 3D-printed cardiac models to construct pericardiocentesis simulators [6,7]. While these models are innovative, 3D-printed models are difficult to attain in low-resource settings. The pericardiocentesis model described in this report has the advantage of construction from readily available materials in a low-resource setting. In addition, this model was made interactive with the help of a live volunteer to engage trainees. The addition of a live volunteer is not limited to this model and maybe a beneficial component for any future pericardiocentesis simulations.

The thoracostomy model also demonstrated several unique advantages in comparison to other simulation models. Walsh et al., Netto et al., and O’Connell et al. incorporated animal chest walls in their low-cost thoracostomy models [8-10]. Garland et al. utilized headsets, cameras for telesimulation, and a 3D-printed chest wall for their thoracostomy model [15]. While all of the aforementioned models have several benefits for effective simulation training, they may not be possible to develop and use in certain cultural and low-resource contexts. The thoracostomy model described in this report was made entirely of plastic- and plant-based materials and required minimal technical expertise to construct. We also used readily available cell phones and locally sourced materials as audio-visual equipment. Notably, our innovative use of mobile phones and live volunteers to engage trainees gave both models heightened fidelity with no increase in cost. Future iterations of this model could involve instructors at remote sites, thereby making these simulations accessible to trainees in remote settings [15].

There are a few limitations of the simulators and associated simulations that were developed for these workshops. The primary of these is that of the limited clinical immersion offered by the simulators themselves. The use of low-cost and readily available materials impacted the haptic feedback available to participants and consequently impacted the recreation of true-to-life instrument handling required to complete the various procedures. Given the setting and goals of our work, maintaining a low overhead cost was important. However, future iterations of these workshops will strive to enhance the clinical immersion offered to trainees by using different materials that increase the fidelity of the simulators themselves while balancing cost limitations. A second limitation is the immersive quality of the clinical scenario itself. The incorporation of clinical elements, such as a fluid pump to simulate a heartbeat in the pericardiocentesis simulation or functioning bedside ultrasound for the thoracostomy simulation, will potentially increase learner engagement. Finally, these simulators and workshops served as proof-of-concept evaluations and functionality testing for the simulator designs. At present, our evaluations only test level 1 (learner satisfaction) of Kirkpatrick’s four-level evaluation mode [16]. Future iterations of these workshops will need to be conducted on a larger scale using established training rubrics of simulation skill training to further validate our low-cost simulation models.

## Conclusions

We worked closely with local educators to develop a new high-tech simulation center, conduct a “training-the-trainer” workshop, and develop novel, low-cost, locally sourced hybrid-fidelity training models for ultrasound pericardiocentesis and chest tube placement. Participants in the training-the-trainer workshop and low-cost model workshops reported having positive educational experiences. Our work illustrates how high-cost, high-tech simulation centers and innovative low-cost models are both viable options for improving access to surgical simulation training in low-resource settings. Successful interventions depend on working closely with local leaders and carefully tailoring the approach to meet local instructional needs. Collaboration is key to increasing access to surgical simulations, particularly in low to middle-income countries.
